# The Impact of COVID-19 Vaccine Controversy on Parents’ Perceptions of Routine Vaccinations

**DOI:** 10.7759/cureus.63606

**Published:** 2024-07-01

**Authors:** Ahmad Alzahrani, Waleed A Al-Shehri, Fahad A Alghamdi, Abdulrahman T Almalki, Khalid H Alzaidi, Husain F Alsulaimani, Shadi Tamur, Abdullah M Khayat, Maryam S Aljaid, Sultan Al-Malki

**Affiliations:** 1 Department of Pediatrics, College of Medicine, Taif University, Taif, SAU

**Keywords:** covid-19, pediatric, vaccine hesitancy, routine vaccines, coronavirus vaccine

## Abstract

Background

The COVID-19 pandemic has significantly influenced public perceptions and behaviors related to vaccination. Understanding parental attitudes, knowledge gaps, and vaccination practices post-pandemic is crucial for informing effective public health strategies. This study aimed to investigate parental attitudes, knowledge, and practices toward routine childhood vaccination in the post-COVID-19 era, emphasizing shifts in perspectives and implications for vaccination strategies.

Methodology

A cross-sectional survey was conducted among 498 parents to assess their attitudes, knowledge, and practices regarding vaccination. Data were analyzed using descriptive statistics, chi-square tests, and t-tests where applicable, with p-values <0.05 considered statistically significant.

Results

The study revealed diverse parental attitudes toward vaccination post-COVID-19. While a majority (72.9%) maintained positive attitudes toward vaccination schedules and benefits, concerns regarding vaccine safety and efficacy were noted. Knowledge gaps persisted, with 16.5% strongly agreeing that children’s vaccinations are weak and have no impact on disease prevention. Despite high adherence to vaccination schedules (68.9%), motivations behind vaccine administration were questioned, as 48.2% strongly disagreed that vaccination was solely for regulatory purposes.

Conclusions

Post-COVID-19, parental attitudes toward vaccination have evolved, reflecting increased concerns about safety and efficacy. Addressing knowledge gaps, combating misinformation, and enhancing trust in vaccination programs are imperative. Tailored communication strategies, education campaigns, and policy interventions are essential to promote vaccination acceptance and safeguard public health resilience in the post-pandemic era.

## Introduction

Vaccination is the administration of a vaccine to trigger immunity by activating the immune system to produce defenses against a specific illness. Community immunity begins when a substantial percentage of a population has received a vaccination [1]. The success stories of eradicating smallpox from the world and poliomyelitis from four World Health Organization zones highlight the importance of immunization efforts. They have made significant contributions to reducing the mortality and morbidity associated with several infectious illnesses. The success of vaccination programs is based on a high percentage of vaccine coverage. This directly protects vaccinated individuals and indirectly the entire community by establishing herd immunity and, as a result, decreasing the spread of vaccine-preventable diseases (VPDs) [2].

Even in areas where vaccination rates are high, some parents continue to have various worries and misconceptions about child immunizations even though their efficacy has been demonstrated [3]. Vaccine hesitancy occurs along a spectrum ranging from full acceptance to open refusal of all vaccines, i.e., when certain immunizations are accepted but others are delayed or refused. Factors such as complacency, convenience, and confidence all have an impact on it.

Vaccine-hesitant parents (VHPs) are parents who may deny one or two vaccines but agree to all others, postpone vaccines, or accept vaccines but are unsure [4]. VHPs are critical for understanding and combating vaccination resistance. They are a significantly larger group than those who entirely reject vaccines [5], and they may be more receptive to behavior modification as they seek information about vaccines from their child’s healthcare provider [6,7]. While VHPs play a crucial role in understanding and combating vaccination resistance, it is important to recognize that their significance extends beyond routine immunizations. In the context of the broader vaccination landscape, their attitudes and concerns became even more relevant, especially during the COVID-19 pandemic. Universal COVID-19 immunization is regarded as the most important method for limiting the spread of SARS-CoV-2 and the risk of new emerging variants. The success of vaccination campaigns is dependent, among other factors, on the immunization of large populations of children and adults in low/middle-income countries where SARS-CoV-2 variants of concern have been found. Lack of faith in the vaccination’s safety and effectiveness is the most consistent predictor of parental COVID-19 vaccine hesitancy, followed by distrust of the government and beliefs that children are not vulnerable to the disease. Demographic factors have also been linked to parental acceptance of the COVID-19 vaccination. Lower parental income and education, as well as whether the parent has gotten the COVID-19 immunization, are examples of these [8].

Parental vaccine hesitancy is becoming an increasingly important public health concern in the United States. In March 2020, an assessment of the latest Centers for Disease Control and Prevention National Immunization Survey data found that more than one-third of U.S. children between the ages of 19 and 35 months were not following the recommended early childhood immunization schedule. Furthermore, a 2019 national survey found that approximately one in four parents reported serious concerns about vaccinating their children. Vaccine hesitancy is now associated with a decrease in vaccine coverage and an increase in VPD outbreaks and epidemics in the United States [8].

Freed and co-workers administered online surveys to a nationally representative sample of parents with children under the age of 18 to assess parents’ trust in vaccine information received from various sources. The study’s key finding was that parents trusted the source of vaccine safety information they used. In the questionnaire, parents said they trusted their child’s pediatrician the most (76%), followed by other healthcare providers (26%), government vaccine experts (23%), and relatives and friends (15%) [9,10]. At least 26% of parents expressed some faith in celebrities. Overall, 73% of parents had some faith in other parents who claimed that a vaccine had injured their child. There were also gender differences among the parents. Parents who claimed a vaccine harmed their kids followed celebrity talks, television shows, magazines, and news articles about vaccine safety [10].

The expanded vaccination program in the Kingdom of Saudi Arabia (KSA) began in 1979 and initially included vaccines for diphtheria, tetanus, and pertussis (DTP); poliomyelitis; and tuberculosis before being expanded to include other vaccines [11]. At the time, the legislation supported the practice of linking birth certificate issuance to completion of primary vaccines within the first two years of life. This procedure was altered 10 years ago to associate the vaccination card with school admission at the age of six. The National Immunization Program eliminated neonatal tetanus and polio. Measles, mumps, and rubella are considered to be eradicated by 2020 [12]. According to the Saudi Ministry of Health’s 2021 immunization coverage figures, BCG coverage is 94%, DTP coverage is 97%, and inactivated polio vaccine coverage is 97%. However, high immunization coverage rates do not reflect a great trust in the vaccines. Many parents’ decisions to immunize their children are likely influenced by the legal requirements of a complete vaccination card for a child’s admission to school in various countries. Furthermore, even parents who vaccinate their children have ignorance and concerns about vaccinations; consequently, initiatives aimed at modifying their views will likely reduce their level of reluctance [13].

This study aimed to investigate parental attitudes, knowledge, and practices regarding vaccination in the post-COVID-19 era in Mecca, KSA, focusing on the impact of the COVID-19 vaccine controversy on their beliefs and behaviors about routine vaccinations.

## Materials and methods

Study design

A cross-sectional study design was employed to investigate parental attitudes, knowledge, and practices about vaccination in the post-COVID-19 era. The study was conducted over a period of six months, from January to June 2023, in the Mecca region of KSA, encompassing both urban and rural settings. This setting was chosen to ensure a representative sample of parents from different socioeconomic backgrounds and geographic locations, enhancing the generalizability of the study findings.

Participants

The participantsincluded parents or legal guardians aged 18 years and above with at least one child aged 0-18 years residing in the study area. Individuals who did not meet the inclusion criteria, non-residents of the study area, and those unwilling to participate or provide informed consent were excluded. A total of 498 participants were included in the study, who were selected through convenience sampling to ensure the feasibility and accessibility of data collection.

Data collection

A structured questionnaire was developed as the primary data collection tool, comprising both closed-ended and Likert-scale questions. The questionnaire was designed based on a comprehensive literature review and expert input to ensure content validity and relevance to the study objectives. It included sections on parental attitudes, knowledge, practices, demographic information, and sources of information related to vaccination.

Data were collected through face-to-face interviews conducted by trained research assistants. The interviews were conducted in a confidential and non-coercive manner, ensuring participant privacy and autonomy. Interviewers followed a standardized protocol to maintain consistency and accuracy in data collection across all participants.

Data management and analysis

Data obtained from the interviews were entered into a database using SPSS version 26 software (IBM Corp., Armonk, NY, USA). The database was organized with appropriate variables and coding to facilitate data analysis. Data cleaning and validation were performed to identify and rectify any inconsistencies or errors in the dataset.

Descriptive statistics were used to summarize the sociodemographic characteristics of the participants. Inferential statistics, including chi-square tests, were employed to examine associations between sociodemographic factors and attitudes, knowledge, and practices regarding vaccination. The significance level was set at p-values <0.05 for all statistical tests.

Ethical considerations

The study adhered to ethical guidelines, and ethical approval was obtained from the Scientific Research Ethics Committee, Taif University, KSA (approval number: 44-366, Date: 13-06-2023). Informed consent was obtained from each participant before data collection, emphasizing confidentiality and the voluntary nature of participation.

## Results

Participant characteristics

Table 1 provides a comprehensive overview of the characteristics of the study participants and their sources of knowledge about vaccines. The study included 498 participants, representing diverse sociodemographic parameters. The age distribution showed that most participants were aged 30-34 years (82 participants, 16.5%), 35-39 years (76 participants, 15.3%), and 40-44 years (92 participants, 18.5%). Only 10 (2%) participants were under 20 years old, and one (0.2%) participant was over 65 years old.

**Table 1:** Characteristics of participants and sources of knowledge about vaccines (n = 498).

Parameter		Frequency (%)
Age, years	<20	10 (2%)
	>65	1 (0.2%)
	20–24	23 (4.6%)
	25–29	45 (9%)
	30–34	82 (16.5%)
	35–39	76 (15.3%)
	40–44	92 (18.5%)
	45–49	93 (18.7%)
	50–54	50 (10%)
	55–59	21 (4.2%)
	60–64	5 (1%)
Educational level	Primary education	23 (4.6%)
	University degree	306 (61.4%)
	Secondary education	72 (14.5%)
	Diploma	39 (7.8%)
	Postgraduate degree	43 (8.6%)
	Intermediate education	15 (3%)
Number of offspring	Less than three	231 (46.4%)
	Three or more	267 (53.6%)
Average family monthly income, SAR	10,000–19,000	215 (43.2%)
	20,000–29,000	93 (18.7%)
	30,000–39,000	32 (6.4%)
	40,000 or more	17 (3.4%)
	Less than 10,000	141 (28.3%)
Occupation	Businessperson	22 (4.4%)
	Not employed	184 (36.9%)
	Healthcare provider	25 (5%)
	Governmental sector	221 (44.4%)
	Private sector	46 (9.2%)
City of residency in Mecca	Adum	6 (1.2%)
	Jumum	7 (1.4%)
	Khurma	2 (0.4%)
	Taif	268 (53.8%)
	Ardyat	5 (1%)
	Konfuda	4 (0.8%)
	Kamel	2 (0.4%)
	Laith	3 (0.6%)
	Moya	2 (0.4%)
	Bahra	4 (0.8%)
	Terba	11 (2.2%)
	Jeddah	81 (16.3%)
	Khales	3 (0.6%)
	Rabeg	2 (0.4%)
	Rania	1 (0.2%)
	Mecca	92 (18.5%)
	Messan	5 (1%)
Age of your youngest offspring	Less than 1 year	79 (15.9%)
	1-4 years	166 (33.3%)
	5-8 years	110 (22.1%)
	9-11 years	65 (13.1%)
	12 years	78 (15.7%)
Educational level of the youngest offspring	Primary education	210 (42.2%)
	Pre-school	28 (5.6%)
	Kindergarten	48 (9.6%)
	Not studying	212 (42.6%)
Source of information regarding vaccines	Internet and social media	122 (24.5%)
	Medical websites and journals	79 (15.9%)
	Advice from healthcare providers	233 (46.8%)
	Advice from friends and family	64 (12.9%)
Do you know anyone who suffered complications from vaccines?	No	376 (75.5%)
	Yes	122 (24.5%)

Regarding educational levels, a significant proportion held university degrees, with 306 (61.4%) participants, followed by those with secondary education (72 participants, 14.5%) and diploma holders (39 participants, 7.8%). In terms of family size, 231 (46.4%) participants had fewer than three children, while 267 (53.6%) had three or more children. The majority of participants reported an average family income between 10,000 and 19,000 SAR, with 215 (43.2%) participants. Occupationally, most participants were employed in the governmental sector (221 participants, 44.4%), followed by those not employed (184 participants, 36.9%).

Geographically, Taif had the highest representation, with 268 (53.8%) participants, followed by Jeddah (81 participants, 16.3%) and Mecca (92 participants, 18.5%). Participants’ youngest offspring were mostly aged one to four years (166 participants, 33.3%) and less than one year (79 participants, 15.9%). The majority of the youngest offspring were in primary education (210 participants, 42.2%). The most common sources of information about vaccines were healthcare providers (233 participants, 46.8%) and the internet/social media (122 participants, 24.5%).

Parental knowledge and attitudes

Table 2 presents parents’ knowledge of children’s vaccination. A majority strongly agreed that following the vaccination schedule is beneficial for health (363 participants, 72.9%), that multiple vaccinations are beneficial (318 participants, 63.9%), and that many diseases prevented by vaccinations can be severe (312 participants, 62.7%). Furthermore, 330 (66.3%) participants strongly agreed that vaccinations help prevent the spread of infectious diseases.

**Table 2:** Knowledge of parents toward children’s vaccination (n = 498).

Parameter		Frequency (%)
Following the child’s vaccination schedule is beneficial for their health	Strongly agree	363 (72.9%)
	Agree	72 (14.5%)
	Strongly disagree	21 (4.2%)
	Disagree	16 (3.2%)
	Neutral	26 (5.2%)
A healthy child receiving multiple vaccinations is beneficial for them	Strongly agree	318 (63.9%)
	Agree	93 (18.7%)
	Strongly disagree	19 (3.8%)
	Disagree	30 (6%)
	Neutral	38 (7.6%)
Many diseases prevented by vaccinations can be severe	Strongly agree	312 (62.7%)
	Agree	113 (22.7%)
	Strongly disagree	13 (2.6%)
	Disagree	24 (4.8%)
	Neutral	36 (7.2%)
Vaccinations help prevent the spread of infectious diseases and epidemics	Strongly agree	330 (66.3%)
	Agree	103 (20.7%)
	Strongly disagree	15 (3%)
	Disagree	20 (4%)
	Neutral	30 (6%)
Children’s vaccinations are weak and have no impact on disease prevention	Strongly agree	54 (10.8%)
	Agree	39 (7.8%)
	Strongly disagree	239 (48%)
	Disagree	109 (21.9%)
	Neutral	57 (11.4%)
The risks of vaccinations outweigh their benefits	Strongly agree	49 (9.8%)
	Agree	45 (9%)
	Strongly disagree	218 (43.8%)
	Disagree	115 (23.1%)
	Neutral	71 (14.3%)
I trust the information I receive about the effectiveness and benefits of vaccinations	Strongly agree	244 (49%)
	Agree	144 (28.9%)
	Strongly disagree	17 (3.4%)
	Disagree	34 (6.8%)
	Neutral	59 (11.8%)
In our current time, most vaccinations are for uncommon diseases	Strongly agree	94 (18.9%)
	Agree	123 (24.7%)
	Strongly disagree	97 (19.5%)
	Disagree	92 (18.5%)
	Neutral	92 (18.5%)
Knowledge score, % (mean ± SD)	74.3 ± 24.8	
Knowledge category	Good knowledge	391 (78.5%)
	Poor knowledge	107 (21.5%)

However, there was some skepticism, with 54 (10.8%) participants strongly agreeing that children’s vaccinations are weak and have no impact on disease prevention. Regarding the perceived risks versus benefits of vaccinations, 239 (48%) participants strongly disagreed that vaccinations are weak, and 218 (43.8%) participants strongly disagreed that the risks outweigh the benefits. Trust in the effectiveness and benefits of vaccinations was high, with 244 (49%) participants strongly agreeing and 144 (28.9%) participants agreeing. Opinions were divided on the prevalence of vaccinations for uncommon diseases, with 94 (18.9%) participants strongly agreeing and 97 (19.5%) participants strongly disagreeing.

The overall knowledge score averaged 74.3 ± 24.8%, categorizing 391 (78.5%) participants as having good knowledge and 107 (21.5%) as having poor knowledge regarding children’s vaccinations.

Parental attitudes toward vaccination

Table 3 presents an analysis of parents’ attitudes toward children’s vaccination. The data reflect diverse attitudes and concerns. A majority (57%) strongly disagreed with being hesitant about adhering to the vaccination schedule, and 211 (42.4%) participants strongly disagreed that it is better to let the child’s immune system fight instead of vaccinating.

**Table 3:** Attitude of parents toward children’s vaccination (n = 498).

Parameter		Frequency (%)
I am hesitant about adhering to the vaccination schedule for my child/children	Strongly agree	58 (11.6%)
	Agree	56 (11.2%)
	Strongly disagree	284 (57%)
	Disagree	66 (13.3%)
	Neutral	34 (6.8%)
It is better to let the child’s immune system fight instead of giving them vaccines	Strongly agree	60 (12%)
	Agree	71 (14.3%)
	Strongly disagree	211 (42.4%)
	Disagree	100 (20.1%)
	Neutral	56 (11.2%)
My child/children are in excellent health, and there is no need to adhere to the vaccination schedule	Strongly agree	59 (11.8%)
	Agree	38 (7.6%)
	Strongly disagree	267 (53.6%)
	Disagree	94 (18.9%)
	Neutral	40 (8%)
It is better not to have the child receive many vaccinations at once	Strongly agree	168 (33.7%)
	Agree	129 (25.9%)
	Strongly disagree	66 (13.3%)
	Disagree	50 (10%)
	Neutral	85 (17.1%)
I am concerned that my child may suffer severe side effects due to any of the vaccinations	Strongly agree	106 (21.3%)
	Agree	125 (25.1%)
	Strongly disagree	93 (18.7%)
	Disagree	97 (19.5%)
	Neutral	77 (15.5%)
I am concerned that any dose of vaccination is unsafe for my child	Strongly agree	106 (21.3%)
	Agree	108 (21.7%)
	Strongly disagree	134 (26.9%)
	Disagree	80 (16.1%)
	Neutral	70 (14.1%)
I am concerned that any dose of vaccination does not protect against the disease and is of no benefit	Strongly agree	82 (16.5%)
	Agree	87 (17.5%)
	Strongly disagree	171 (34.3%)
	Disagree	89 (17.9%)
	Neutral	69 (13.9%)
I can discuss my concerns and fears about vaccinations with my child’s pediatrician	Strongly agree	307 (61.6%)
	Agree	117 (23.5%)
	Strongly disagree	18 (3.6%)
	Disagree	26 (5.2%)
	Neutral	30 (6%)
I believe that the coronavirus vaccine is effective and has an impact on increasing my child’s immunity against the virus	Strongly agree	110 (22.1%)
	Agree	97 (19.5%)
	Strongly disagree	80 (16.1%)
	Disagree	85 (17.1%)
	Neutral	126 (25.3%)
I believe that the coronavirus vaccine is important to protect other children from the virus spreading among them and their families	Strongly agree	135 (27.1%)
	Agree	113 (22.7%)
	Strongly disagree	70 (14.1%)
	Disagree	66 (13.3%)
	Neutral	114 (22.9%)
I believe that the coronavirus vaccine has more serious side effects than benefits	Strongly agree	123 (24.7%)
	Agree	115 (23.1%)
	Strongly disagree	62 (12.4%)
	Disagree	84 (16.9%)
	Neutral	114 (22.9%)
I believe that the coronavirus vaccine is just marketing for pharmaceutical companies	Strongly agree	120 (24.1%)
	Agree	75 (15.1%)
	Strongly disagree	91 (18.3%)
	Disagree	79 (15.9%)
	Neutral	133 (26.7%)
Do you feel that the controversy surrounding the coronavirus vaccine has increased your concerns about other vaccinations?	Maybe	155 (31.1%)
	No	153 (30.7%)
	Yes	190 (38.2%)
Attitude score, % (mean ± SD)	48.7 ± 25.9	
Attitude category	Positive attitude	232 (46.6%)
	Negative attitude	266 (53.4%)

Regarding the necessity of vaccinations, 267 (53.6%) participants strongly disagreed that their child’s good health negates the need for vaccinations, while 168 (33.7%) participants strongly agreed that it is better not to receive many vaccinations at once. Concerns about vaccine safety were present, with 106 (21.3%) participants strongly agreeing about severe side effects, whereas 134 (26.9%) participants strongly disagreed that any dose of vaccination is ineffective.

Attitudes toward the COVID-19 vaccine were mixed. While 135 (27.1%) participants strongly agreed on its importance in protecting against the virus, 123 (24.7%) participants strongly agreed that it has more serious side effects than benefits. The overall attitude score was 48.7 ± 25.9%, with 232 (46.6%) participants categorized as having a positive attitude and 266 (53.4%) as having a negative attitude toward vaccinations.

Parental practices toward vaccination

Table 4 assesses parental practices toward children’s vaccination. The majority, 343 (68.9%) participants, reported not delaying any dose of their child’s vaccine schedule for reasons other than illness or allergy. Additionally, 385 (77.3%) participants strongly agreed that they would ensure their children receive all scheduled vaccinations. However, 380 (76.3%) participants reported that their youngest child had not received any doses of the COVID-19 vaccine, while 234 (47%) participants stated that their other children had received the vaccine.

**Table 4:** Practice of parents toward children’s vaccination (n = 498).

Parameter		Frequency (%)
Have you delayed any dose of your child’s vaccine schedule for reasons other than illness or allergy?	No	343 (68.9%)
	Yes	155 (31.1%)
If I had another child now, I would ensure they receive all doses from their vaccination schedule	Strongly agree	385 (77.3%)
	Agree	58 (11.6%)
	Strongly disagree	10 (2%)
	Disagree	16 (3.2%)
	Neutral	29 (5.8%)
The only reason my child receives vaccinations is for them to enter childcare or school	Strongly agree	69 (13.9%)
	Agree	49 (9.8%)
	Strongly disagree	240 (48.2%)
	Disagree	98 (19.7%)
	Neutral	42 (8.4%)
Has your youngest child received any doses of the coronavirus vaccine?	No	380 (76.3%)
	Yes	118 (23.7%)
Have your other children received any doses of the coronavirus vaccine?	No	156 (31.3%)
	No other children	108 (21.7%)
	Yes	234 (47%)
I give the coronavirus vaccine to my child/children only because it is mandatory to obtain	Strongly agree	121 (24.3%)
	Agree	104 (20.9%)
	Strongly disagree	102 (20.5%)
	Disagree	66 (13.3%)
	Neutral	105 (21.1%)
Practice score, % (mean ± SD)	55.9 ± 21.8	
Practice	Good practice	209 (42%)
	Poor practice	289 (58%)

Regarding motivations for administering the COVID-19 vaccine, 240 (48.2%) participants strongly disagreed that the only reason was for childcare or school entry. The practice score averaged 55.9 ± 21.8%, with 209 (42%) participants categorized as having good practices and 289 (58%) as having poor practices toward children’s vaccinations.

Table 5 examines the association between the COVID-19 vaccine controversy and concerns about other vaccinations. No statistically significant association was found between age, education level, number of children, family income, or occupation and increased concerns about vaccinations. However, significant associations were observed with the educational level of the youngest offspring (p = 0.032) and sources of information (p = 0.006). Participants relying on the internet and social media showed higher concerns compared to those relying on medical websites, healthcare providers, or friends and family. Additionally, knowing someone who suffered complications from vaccines significantly increased concerns about other vaccinations (p < 0.0001).

**Table 5:** Effect of the COVID-19 vaccine controversy on raising concerns about other vaccinations in association with sociodemographic factors and source of information (n = 498).

Parameter		COVID-19 vaccine controversy increased concerns about other vaccinations			χ^2^	P-value
		Maybe	No	Yes		
Age, years	<20	3 (30%)	3 (30%)	4 (40%)	22.6	0.309
	>65	0 (0%)	1 (100%)	0 (0%)		
	20–24	8 (34.8%)	5 (21.7%)	10 (43.5%)		
	25–29	18 (40%)	11 (24.4%)	16 (35.6%)		
	30–34	28 (34.1%)	29 (35.4%)	25 (30.5%)		
	35–39	19 (25%)	25 (32.9%)	32 (42.1%)		
	40–44	25 (27.2%)	24 (26.1%)	43 (46.7%)		
	45–49	25 (26.9%)	32 (34.4%)	36 (38.7%)		
	50–54	24 (48%)	12 (24%)	14 (28%)		
	55–59	3 (14.3%)	9 (42.9%)	9 (42.9%)		
	60–64	2 (40%)	2 (40%)	1 (20%)		
Educational level	Primary education	7 (30.4%)	9 (39.1%)	7 (30.4%)	12.3	0.267
	University degree	89 (29.1%)	90 (29.4%)	127 (41.5%)		
	Secondary education	26 (36.1%)	18 (25%)	28 (38.9%)		
	Diploma	14 (35.9%)	10 (25.6%)	15 (38.5%)		
	Postgraduate degree	15 (34.9%)	19 (44.2%)	9 (20.9%)		
	Intermediate education	4 (26.7%)	7 (46.7%)	4 (26.7%)		
Number of offspring	Less than three	71 (30.7%)	69 (29.9%)	91 (39.4%)	0.3	0.862
	Three or more	84 (31.5%)	84 (31.5%)	99 (37.1%)		
Average family monthly income, SAR	10,000–19,000	68 (31.6%)	58 (27%)	89 (41.4%)	8.7	0.365
	20,000–29,000	29 (31.2%)	33 (35.5%)	31 (33.3%)		
	30,000–39,000	7 (21.9%)	10 (31.3%)	15 (46.9%)		
	40,000 or more	4 (23.5%)	9 (52.9%)	4 (23.5%)		
	Less than 10,000	47 (33.3%)	43 (30.5%)	51 (36.2%)		
Occupation	Businessman	9 (40.9%)	3 (13.6%)	10 (45.5%)	5.6	0.692
	Not employed	61 (33.2%)	58 (31.5%)	65 (35.3%)		
	Healthcare provider	7 (28%)	10 (40%)	8 (32%)		
	Governmental sector	66 (29.9%)	67 (30.3%)	88 (39.8%)		
	Private sector	12 (26.1%)	15 (32.6%)	19 (41.3%)		
City of residency in Mecca	Adum	3 (50%)	2 (33.3%)	1 (16.7%)	42.1	0.109
	Jumum	2 (28.6%)	1 (14.3%)	4 (57.1%)		
	Khurma	1 (50%)	1 (50%)	0 (0%)		
	Taif	93 (34.7%)	71 (26.5%)	104 (38.8%)		
	Ardyat	2 (40%)	1 (20%)	2 (40%)		
	Konfuda	1 (25%)	0 (0%)	3 (75%)		
	Kamel	0 (0%)	1 (50%)	1 (50%)		
	Laith	2 (66.7%)	1 (33.3%)	0 (0%)		
	Moya	1 (50%)	0 (0%)	1 (50%)		
	Bahra	0 (0%)	0 (0%)	4 (100%)		
	Terba	3 (27.3%)	4 (36.4%)	4 (36.4%)		
	Jeddah	27 (33.3%)	22 (27.2%)	32 (39.5%)		
	Khales	0 (0%)	1 (33.3%)	2 (66.7%)		
	Rabeg	1 (50%)	0 (0%)	1 (50%)		
	Rania	0 (0%)	0 (0%)	1 (100%)		
	Mecca	18 (19.6%)	45 (48.9%)	29 (31.5%)		
	Messan	1 (20%)	3 (60%)	1 (20%)		
Age of the youngest offspring	12 years	18 (23.1%)	27 (34.6%)	33 (42.3%)	8.1	0.420
	5–8 years	33 (30%)	39 (35.5%)	38 (34.5%)		
	9–11 years	20 (30.8%)	23 (35.4%)	22 (33.8%)		
	Less than 1 year	29 (36.7%)	17 (21.5%)	33 (41.8%)		
	1–4 years	55 (33.1%)	47 (28.3%)	64 (38.6%)		
Educational level of the youngest offspring	Primary education	51 (24.3%)	76 (36.2%)	83 (39.5%)	13.8	0.032
	Preschool	9 (32.1%)	9 (32.1%)	10 (35.7%)		
	Kindergarten	19 (39.6%)	17 (35.4%)	12 (25%)		
	Not studying	76 (35.8%)	51 (24.1%)	85 (40.1%)		
Source of information regarding vaccines	Internet and social media	34 (27.9%)	26 (21.3%)	62 (50.8%)	18.3	0.006
	Medical websites and journals	28 (35.4%)	23 (29.1%)	28 (35.4%)		
	Advice from healthcare providers	69 (29.6%)	89 (38.2%)	75 (32.2%)		
	Advice from friends and family	24 (37.5%)	15 (23.4%)	25 (39.1%)		
Do you know anyone who suffered complications from vaccines?	No	129 (34.3%)	122 (32.4%)	125 (33.2%)	16.2	0.000
	Yes	26 (21.3%)	31 (25.4%)	65 (53.3%)		

## Discussion

The COVID-19 pandemic has profoundly impacted global healthcare systems and public health perceptions, including attitudes toward vaccination. Understanding how parental perspectives have evolved in the aftermath of the pandemic is crucial for shaping effective vaccination strategies [1-3]. This discussion delves into our study’s findings regarding parental attitudes, knowledge, and practices toward vaccination, emphasizing post-COVID-19 stances and implications.

Our study revealed notable shifts in parental attitudes toward vaccination post-COVID-19. While a significant proportion maintained positive attitudes toward vaccination schedules and benefits, there was an evident increase in concerns regarding vaccine safety and efficacy. For instance, 123 (24.7%) strongly agreed that the COVID-19 vaccine has more serious side effects than benefits, reflecting heightened apprehensions following pandemic-related vaccine developments.

This shift is understandable given the rapid development and emergency use authorizations of COVID-19 vaccines, which may have raised questions about long-term safety and efficacy [9]. The emergence of vaccine hesitancy clusters post-pandemic highlights the need for targeted education campaigns, transparent communication, and continuous monitoring of vaccine sentiment to address evolving concerns effectively [10,13,14].

Despite widespread access to information during the pandemic, our study identified persistent knowledge gaps and misinformation regarding vaccines. While a majority recognized the importance of vaccination, misconceptions about vaccine risks and benefits persisted. For instance, 82 (16.5%) strongly agreed that children’s vaccinations are weak and have no impact on disease prevention, indicating persistent myths that vaccines are ineffective or unnecessary [15-17].

The proliferation of misinformation, especially on social media platforms, has contributed to vaccine hesitancy and skepticism. Post-pandemic, combating misinformation and promoting evidence-based information remain critical challenges. Health authorities and stakeholders must prioritize targeted communication strategies, leveraging credible sources and engaging with communities to address misconceptions effectively [18-20].

The controversies surrounding COVID-19 vaccines have had a significant impact on broader vaccination attitudes and practices. Our study found that 190 (38.2%) agreed that the controversy surrounding the COVID-19 vaccine increased their concerns about other vaccinations. This ripple effect underscores the interconnectedness of vaccine perceptions and the need for nuanced approaches to address hesitancy comprehensively [20-22].

Policy implications post-COVID-19 include enhancing vaccine access, promoting vaccine literacy, and fostering trust in immunization programs. Tailored interventions targeting specific hesitancy drivers, such as safety concerns and misinformation, can bolster vaccine acceptance. Additionally, integrating vaccination education into school curricula and leveraging community influencers can facilitate positive behavioral shifts toward vaccination [22,23]. Participants relying on the internet/social media exhibited higher levels of concern, highlighting the influence of online discourse and the imperative to counter misinformation ecosystems effectively. Collaborative efforts among healthcare providers, social media platforms, and public health agencies are essential to promote accurate vaccine information and combat vaccine hesitancy [22-24].

While our study indicated high adherence to vaccination schedules, concerns about the motivation behind vaccine administration were raised. Specifically, 240 (48.2%) strongly disagreed that the only reason their child receives vaccinations is for them to enter childcare or school. This signals a broader recognition of vaccination’s role beyond regulatory requirements, emphasizing the need for comprehensive vaccination strategies encompassing health promotion and disease prevention [20,23,25]. The association between sources of vaccine information and increased concerns about other vaccinations is noteworthy [25-27].

Comparing our findings with global trends reveals both convergences and divergences in post-pandemic vaccination stances. Globally, there has been a surge in vaccine hesitancy, driven by safety concerns, distrust in authorities, and misinformation. However, successful vaccination campaigns, transparent communication, and proactive engagement have mitigated hesitancy in some regions [15-18,20,26,27].

Future research directions should prioritize longitudinal studies tracking post-COVID-19 vaccination attitudes and behaviors. Long-term monitoring of vaccine sentiment, evaluating the effectiveness of interventions, and exploring cultural nuances in vaccine acceptance are crucial. Collaborative research efforts and data sharing across regions can inform evidence-based strategies to address vaccine hesitancy comprehensively.

While the study highlights important aspects of vaccination behavior, it is crucial to acknowledge certain limitations that may impact the generalizability and validity of the findings. First, the reliance on a cross-sectional survey design limits the ability to establish causal relationships between variables, as it captures data at a single point in time. Longitudinal studies may provide a more nuanced understanding of how parental attitudes toward vaccination evolve over time. Additionally, the study sample of 498 parents may not be fully representative of the broader population, potentially introducing selection bias. Moreover, the use of self-reported measures for assessing attitudes and knowledge leaves room for response bias and social desirability effects, where participants may provide answers they believe are socially acceptable rather than reflecting their true beliefs. It is also important to consider the cultural and regional differences that could influence parental perspectives on vaccination, which were not thoroughly explored in this study. Future research endeavors could benefit from addressing these limitations to offer a more comprehensive understanding of vaccination behavior post-pandemic and enhance the effectiveness of public health initiatives.

## Conclusions

The post-COVID-19 landscape has reshaped parental attitudes, knowledge, and practices regarding vaccination. While challenges such as misinformation and safety concerns persist, opportunities exist to strengthen vaccination advocacy, communication, and policy frameworks. Addressing evolving concerns, leveraging digital health technologies, and fostering community partnerships are integral to promoting vaccination acceptance and safeguarding public health resilience in the post-pandemic era.

The controversies surrounding COVID-19 vaccinations have also had a ripple effect on broader vaccination attitudes, emphasizing the interconnected nature of vaccine perceptions and the need for nuanced strategies to tackle hesitancy comprehensively. Recognizing the influence of different sources of vaccine information on public concerns, collaborative efforts among healthcare providers, social media platforms, and public health agencies are crucial to disseminating accurate information and addressing vaccine hesitancy effectively. Furthermore, while our study highlighted strong adherence to vaccination schedules, it also raised concerns about the underlying motivations for vaccine administration, indicating a growing awareness of the broader health benefits of vaccinations beyond regulatory compliance. Moving forward, comprehensive vaccination strategies that encompass health promotion and disease prevention will play a pivotal role in shaping effective vaccination practices and ensuring public health resilience in the face of evolving challenges.

## Appendices

**Table 6:** Survey questions and responses.

Question	Answers
Age	<30, 30–40, >40
Education level	Secondary school, Diploma, Bachelor, Postgraduate
Number of children under your custody	>3, <3
Average monthly household income	<10,000 SAR, 10,000–19,000, 20,000–29,000, 30,000–39,000, >40,000
Current job	Healthcare provider, Government sector employee, Private sector employee, Personal job, I don’t work currently
Place of residence	(Fill in the blank)
Age of youngest child	1–4 years, 5–8 years, 9–11 years, 12 years or older
Educational level of the youngest child	Not studying, Kindergarten, Primary school
Sources of information about vaccines	Internet or social media, Advice from doctors or medical staff, Medical websites or articles, Friends or family advice
Have you delayed any vaccination for any reason besides sickness or allergy?	Yes, No
Following a vaccination plan is good for the child’s health	Totally agree, Somewhat agree, Neutral, Somewhat disagree, Totally disagree
If I had another child, I would make sure he/she receives all doses from his/her vaccination plan	Totally agree, Somewhat agree, Neutral, Somewhat disagree, Totally disagree
I am hesitant about sticking to my child’s/children’s vaccination schedule	Totally agree, Somewhat agree, Neutral, Somewhat disagree, Totally disagree
It is good for a healthy child to receive many vaccinations	Totally agree, Somewhat agree, Neutral, Somewhat disagree, Totally disagree
Many of the diseases that vaccinations prevent are severe	Totally agree, Somewhat agree, Neutral, Somewhat disagree, Totally disagree
It is better to let the child’s immune system fight the disease rather than giving the vaccine	Totally agree, Somewhat agree, Neutral, Somewhat disagree, Totally disagree
Vaccinations help prevent the spread of infectious diseases and epidemics	Totally agree, Somewhat agree, Neutral, Somewhat disagree, Totally disagree
Children’s vaccinations are weak and have no effect on disease prevention	Totally agree, Somewhat agree, Neutral, Somewhat disagree, Totally disagree
My child/children are in excellent health and there is no need to follow the vaccination schedule	Totally agree, Somewhat agree, Neutral, Somewhat disagree, Totally disagree
It is better for the child not to have many vaccinations at one time	Totally agree, Somewhat agree, Neutral, Somewhat disagree, Totally disagree
I am concerned that any dose of immunization may not be safe for my child	Totally agree, Somewhat agree, Neutral, Somewhat disagree, Totally disagree
I am concerned that any dose of vaccination will not prevent the disease and will be of no benefit	Totally agree, Somewhat agree, Neutral, Somewhat disagree, Totally disagree
The risks of vaccinations outweigh the benefits	Totally agree, Somewhat agree, Neutral, Somewhat disagree, Totally disagree
Do you know anyone who has had any adverse reactions to a vaccination?	Yes, No
The only reason for my child to get immunized is to get him into day-care or school	Totally agree, Somewhat agree, Neutral, Somewhat disagree, Totally disagree
I trust the information I receive about the effectiveness and benefits of vaccinations	Totally agree, Somewhat agree, Neutral, Somewhat disagree, Totally disagree
I can discuss my fears and concerns about vaccinations with my pediatrician	Totally agree, Somewhat agree, Neutral, Somewhat disagree, Totally disagree
Nowadays most vaccinations are for uncommon diseases	Totally agree, Somewhat agree, Neutral, Somewhat disagree, Totally disagree
Has your youngest child received any vaccine doses against the COVID-19 virus?	Yes, No
Did the rest of your children get any doses of the COVID-19 vaccine	Yes, No, I don't have other children
I believe that the COVID-19 vaccine is and has an effect in increasing my child’s immunity against the virus	Totally agree, Somewhat agree, Neutral, Somewhat disagree, Totally disagree
I believe that the COVID-19 vaccine is important to protect other children from spreading the virus between them and their parents	Totally agree, Somewhat agree, Neutral, Somewhat disagree, Totally disagree
I only give the COVID-19 vaccine to my child/children because it is mandatory to get it	Totally agree, Somewhat agree, Neutral, Somewhat disagree, Totally disagree
I believe that the COVID-19 vaccine has more serious side effects than benefits	Totally agree, Somewhat agree, Neutral, Somewhat disagree, Totally disagree
I believe that the COVID-19 vaccine is just a way of marketing for drug manufacturers	Totally agree, Somewhat agree, Neutral, Somewhat disagree, Totally disagree
Do you feel that the COVID-19 vaccine controversy has increased your concerns about other vaccinations?	Yes, No, Maybe
